# Viral evolution sustains a dengue outbreak of enhanced severity

**DOI:** 10.1080/22221751.2021.1899057

**Published:** 2021-03-27

**Authors:** Catherine Inizan, Marine Minier, Matthieu Prot, Olivia O’Connor, Carole Forfait, Sylvie Laumond, Ingrid Marois, Antoine Biron, Ann-Claire Gourinat, Marie-Amélie Goujart, Elodie Descloux, Anavaj Sakuntabhai, Arnaud Tarantola, Etienne Simon-Lorière, Myrielle Dupont-Rouzeyrol

**Affiliations:** a URE Dengue and Arboviruses, Institut Pasteur in New Caledonia, Institut Pasteur International Network, Nouméa, New Caledonia; b Evolutionary genomics of RNA viruses, Institut Pasteur, Paris, France; c Health Authorities (DASS), Nouméa, New Caledonia; d Internal medicine and infectious diseases department, Territorial Hospital Center (CHT), Dumbéa, New Caledonia; e Microbiology laboratory, Territorial Hospital Center (CHT), Dumbéa, New Caledonia; f Functional genetics of infectious diseases Unit, Institut Pasteur, Paris, France; g CNRS UMR2000: Génomique évolutive, modélisation et santé (GEMS), Paris, France; h URE Epidemiology, Institut Pasteur in New Caledonia, Institut Pasteur International Network, Nouméa, New Caledonia

**Keywords:** Severe dengue, hepatitis, whole-genome sequencing, viral fitness

## Abstract

Compared to the previous 2013–2014 outbreak, dengue 2016–2017 outbreak in New Caledonia was characterized by an increased number of severe forms associated with hepatic presentations. In this study, we assessed the virological factors associated with this enhanced severity. Whole-genome sequences were retrieved from dengue virus (DENV)-1 strains collected in 2013–2014 and from severe and non-severe patients in 2016–2017. Fitness, hepatic tropism and cytopathogenicity of DENV 2016–2017 strains were compared to those of 2013–2014 strains using replication kinetics in the human hepatic cell line HuH7. Whole-genome sequencing identified four amino acid substitutions specific to 2016–2017 strains and absent from 2013–2014 strains. Three of these mutations occurred in predicted T cell epitopes, among which one was also a B cell epitope. Strains retrieved from severe forms did not exhibit specific genetic features. DENV strains from 2016–2017 exhibited a trend towards reduced replicative fitness and cytopathogenicity *in vitro* compared to strains from 2013–2014. Overall, the 2016–2017 dengue outbreak in New Caledonia was associated with a viral genetic evolution which had limited impact on DENV hepatic tropism and cytopathogenicity. These mutations, however, may have modified DENV strains antigenicity, altering the anti-DENV immune response in some patients, in turn favoring the development of severe forms.

**Trial registration:** ClinicalTrials.gov identifier: NCT04615364.

## Introduction

Dengue infection is an emerging disease caused by dengue virus (DENV), an arbovirus belonging to the *Flaviviridae* family, genus *Flavivirus*. DENV is transmitted to humans by the bite of infected mosquitoes, mainly from the genus *Aedes*. DENVs are divided into four serotypes (DENV-1 to -4), themselves subdivided in genotypes. Infection with one serotype is thought to provide life-long protection from reinfection with the same serotype but does not prevent secondary infection by another serotype. The spectrum of dengue clinical presentations is broad, ranging from asymptomatic to severe, sometimes fatal, infection. Facilitating and standardizing dengue clinical management [1], dengue fever is categorized by WHO 2009 guidelines into dengue without alert signs, dengue with alert signs, which include abdominal pain, persistent vomiting, clinical fluid accumulation and drop in platelet count, and severe dengue. Severe dengue is characterized by severe plasma leakage, severe bleeding and/or severe organ involvement [2].

With an estimated 390 million infections each year, dengue has become the most prevalent human arbovirosis, affecting 128 countries located mainly in tropical and subtropical countries [3]. In New Caledonia (NC), a French island territory of 280,000 inhabitants located in the South Pacific region, the epidemiology of dengue has evolved over the last decade. Dengue outbreaks are becoming increasingly recurrent [4], concurrent with the introduction and circulation of other arboviruses such as Zika and chikungunya viruses [5]. In 2013-2014, NC experienced a massive DENV-1 outbreak of 10,851 reported confirmed or probable cases [6]. In 2016-2017, NC experienced another important DENV outbreak of 5,072 cases [6]. This last dengue outbreak was unusual due to: (i) The co-circulation of DENV-1, -2 and -3 (DENV-1 being responsible for 80.6% of the cases); (ii) Its enhanced severity compared to previous years, leading to 416 hospital admissions (hospital admission rate of 8.2%), and 15 deaths; And (iii) the exceptional rate of hepatic presentations [7]. Major hepatic cytolysis (Aspartate Transminases (AST) and/or Alanine Amino Transferases (ALT) over 1,000 UI/L) was the most frequent severity criterion, accounting for 41.5% of severe cases. Age, comorbidities, presence of at least one alert sign, platelets <30 × 10^9^/L, prothrombin time <60%, AST and/or ALT >10N, previous dengue infection were identified as risk factors to develop severe dengue. Past history of Zika infection was not a risk factor for progression to severe dengue [7].

We compared DENV retrieved during the 2013–2014 and 2016–2017 outbreaks in NC to assess whether any differences in virological factors were associated with the increase in the rate of hepatic presentations.

## Material and methods

### Ethics statement

The study was conducted in compliance with the Declaration of Helsinki principles. Samples collected in 2013–2014 came from patients who did not object to the secondary use of their serum sample for research purposes. This study reusing serum samples received administrative and ethical clearance in France from the “Comité de Protection des Personnes Sud-Est II” (n° ID-RCB 2019-A03114-53, n° CPP 19.12.06.49357) and by the Consultative Ethics Committee of New Caledonia. The study was recorded on Clinicaltrials.gov (ID: NCT04615364). Samples collected in 2016–2017 came from patients who gave their written informed consent as part of this study. This study was approved by the “Comité de Protection des Personnes Ile-de-France I” (n° ID-RCB 2015-A00205-44, CPP n°2015-march-13851 and 2018-january-14793) and by the Consultative Ethics Committee of New Caledonia.

### Patient recruitment and classification

The 2016–2017 sample set was prospectively constituted in the framework of the current study by including consenting patients referring at the Institut Pasteur in New Caledonia or the Territorial Hospital for dengue diagnosis. Written informed consent was received from participants prior to inclusion in the study. Major hemorrhagic signs, shock, thrombocytopenia/bleeding, acute or fulminant hepatitis, diabetes and obesity were documented. Disease severity in patients was assessed using the WHO 2009 guidelines [2]. Blood samples were taken from patients at first referral, between days 0 and 9 post symptom onset. Patients found positive for DENV by qRT-PCR were included in the study. Methods are described elsewehere [8, 9]. The 2013–2014 control set was constituted by retrospectively including 17 DENV biobanked samples. Samples were randomly selected among DENV+ samples in order to cover the entire 2013−2014 epidemic season. All serum samples were stored at −80°C before analysis.

### Laboratory diagnosis

Infecting dengue serotype was determined using qRT-PCR [10]. IgG against dengue and Zika virus were measured by ELISA method (PanBio®, Brisbane, Australia for dengue and and Euroimmun®, Lübeck, Germany for Zika). AST and ALT were titered by spectrophotometry on an Architect C8000 system (Abbott, Lake Forest, USA).

### RNA preparation and generation of RNA libraries

RNA extraction from serum samples was performed using the QiaAmp Viral RNA extraction kit (Qiagen, Hilden, Germany), followed by treatment with Turbo DNase (ThermoFisher, Asnières-sur-Seine, France) to digest contaminating DNA. Host rRNA were depleted from RNA samples using the NEBNext® rRNA Depletion Kit (New England Biolabs, Évry-Courcouronnes, France) as described elsewhere [11]. RNA from selective depletion was used for cDNA synthesis and Illumina library preparation using the Nextera XT kit with dual indexes and sequenced on an Illumina NextSeq500 (75 cycles, paired-end reads) platform.

### RNA sequencing and analysis

Raw paired-end files were processed for removal of Illumina adaptor sequences, trimmed and quality-based filtered using Trimmomatic v0.36 [12]. *De novo* assembly was performed using metaSPAdes v3.12.0 with default parameters [13]. Scaffolds were queried against the NCBI non-redundant protein database [14] using DIAMOND v 0.9.26 [15]. For each sample, the main scaffold corresponded to DENV and no other viruses were identified. Iterative mapping using CLC-assembly-cell v5.1.0 was used to generate full-length or near full-length genomes. Whole-genome sequences are deposited under GenBank Accession Numbers MW315172-MW315195.

### Phylogeny

We downloaded all available complete DENV-1 genomes from the Virus Pathogen Database and Analysis Resource (ViPR) database on 2 November 2019 [16]. We retrieved from GenBank two additional DENV-1 genomes from 2014 generated in the framework of DENV molecular surveillance in NC. Sequence alignment was performed by using MAFFT v7.023 [17]. Maximum-likelihood (ML) phylogenies were inferred using IQ-TREE v1.6.7.2 [18] and branch support was calculated using ultrafast bootstrap approximation with 1,000 replicates [19]. Prior to the tree reconstruction, the ModelFinder application [20], as implemented in IQ-TREE, was used to select the best-fitted nucleotide substitution model.

### Cells and viruses

The *Aedes albopictus* C6/36 cell line was cultured at 28°C in Leibovitz L15 medium (Sigma-Aldrich, Merck, Steinheim, Germany) supplemented with 5% Fetal Calf Serum (FCS, Gibco™, Fisher scientific, Paisley, UK) and 10% Tryptose Phosphate Broth (TPB, Gibco™, Fisher scientific, Paisley, UK). The human hepatocarcinoma HuH7 cell line was cultured at 37°C under 5% CO2 in Dulbecco's Modified Eagle Medium (DMEM, Gibco™, Fisher scientific, Paisley, UK) supplemented with 10% FCS. We recovered DENV from serum samples through infection of C6/36 cells. All final viral stocks were prepared with no more than four passages in C6/36 for 5 days. Supernatants were collected and stored at −80°C.

### Viral titration by immunofluorescent focus assay (IFA)

C6/36 were plated at 2 × 10^4^ cells per well in 96-well plates. Forty-eight hours after seeding, we inoculated wells with serial 10-fold dilutions of the viral stock in Leibovitz L15 medium supplemented with 2% FCS and 10% TPB. After 2 h adsorption, cells were overlaid with 1.6% CarboxyMethylCellulose (VWR, Leuven, Belgium) in L15 medium supplemented with 5% FCS and 5% TPB. Five days after infection, cells were fixed with 4% Paraformaldehyde (Sigma-Aldrich, Switzerland) and permeabilized using 0.5% Triton X-100 (Biorad, Hercules, USA). Cells were then stained for 1 h with anti-dengue complex antibody clone 2H2 (Millipore, Temecula, USA) at 5 µg/mL in PBS supplemented with 2% FCS, followed by 1 h incubation with secondary anti-mouse IgG antibody coupled to Al488 (Invitrogen, Termo Fisher Scientific, Eugene, USA) at 2 µg/mL in PBS supplemented with 2% FCS. Fluorescent infection foci were counted under an inverted fluorescence microscope (DMIL LED, Leica, Wetzlar, Germany).

### Viral replication kinetics in HuH7 hepatic cells

HuH7 were seeded at 2 × 10^5^ cells per well in 12-well plates. Twenty-four hours later, cells were infected with DENV viral stocks at a multiplicity of infection of one. At 24, 48 and 72 h post-infection, supernatants were collected and stored at -80°C in 0.5 M Sucrose (Acros Organics, Geel, Belgium) and 20 mM Hepes buffer (Gibco, Fisher scientific, Paisley, UK) for subsequent titration. Simultaneously, cells were collected using TrypLE-Express (Gibco, Fisher scientific, Grand Island, USA). Cell mortality was assessed using Fixable Viability Dye eFluor 780 (eBioscence, Thermo Fisher Scientific, Carslbad, USA). Cells were then fixed using 4% Paraformaldehyde and stored at 4°C. Fixed cells were permeabilized at 72 h post-infection using 0.5% Triton X-100 and DENV was stained using anti-dengue complex antibody clone 2H2 at 3 µg/mL and secondary anti-mouse IgG Al488 antibody at 2 µg/mL in PBS supplemented with 2% FCS. Mortality and infection rates were determined by flow cytometry on a BD FACSCantoII (BD Biosciences, San Jose, USA). A DENV-1 strain isolated in 2014 in NC and passaged several times in C6/36 cells was used as a positive control. This viral strain yielded nearly 100% infection of HuH7 cells at day 3 post-infection. Percentages of infected cells were normalized by the percentage of infected cells of the positive control at day 3 post-infection. In brief, relative infection percentages of the tested conditions were expressed as a percentage of the infection rate of the positive control at day 3 post-infection: (infection percentage of the tested condition/infection percentage of the positive control at day 3)*100. Viral titers in the supernatants were determined by IFA.

### Epitope prediction

T cell epitopes were predicted using NetCTL 1.2 server, restricting epitopes to the MHC Class I supertype A24, as HLA A*24:02 allele is reported to be the main MHC Class I supertype in New Caledonia (http://www.allelefrequencies.net/). B cell epitopes were predicted using BepiPred 2.0 server with an epitope threshold of 0.5.

### Statistics

Statistical analysis was performed using GraphPad Prism (version 6.1; GraphPad; San Diego, CA; USA). Differences between groups of virus isolates were analysed for statistical significance using a Wilcoxon–Mann–Whitney ranksum test. The *p* value significance threshold was set at 0.05.

## Results

### Characteristics of the sample set

In all, samples from 86 DENV patients were included: 17 biobanked serum samples collected during the 2013–2014 outbreak and 69 patients prospectively included during the 2016–2017 outbreak (Table 1). Blood was collected at a median of two (Interquartile range IQR [1.5; 4]) and three (IQR [2; 5]) days post-symptom onset for patients from 2013–2014 and 2016–2017, respectively. Cases were categorized as severe according to the WHO 2009 classification [2]: no severe cases were included in 2013–2014 and 17/69 (25%) were detected in 2016-2017.

**Table 1.** Demographic data and clinical and biological parameters of the studied populations.

	2013–2014	2016–2017	Total
*All patients*			
*n*	17	69	86
Days since symptom onset at inclusion (median, IQR)	2 [1.5; 4] (2 N/A)	3 [2; 5] (7 N/A)	3 [2; 5] (9 N/A)
WHO 2009 classification [2]			
NS	12/17 (71%)	51/69 (74%)	63/86 (73%)
S	0	17/69 (25%)	17/86 (20%)
N/A	5/17 (29%)	1/69 (1%)	6/86 (7%)
Death	N/A	2/69 (2.9%)	2/86 (2.3%)
Infecting serotype			
DENV-1	14/17 (82%)	53/69 (77%)	67/86 (78%)
DENV-2	0/17 (0%)	12/69 (17%)	12/86 (14%)
DENV-3	3/17 (18%)	4/69 (6%)	7/86 (8%)
DENV-4	0/17 (0%)	0/69 (0%)	0/86 (0%)
Viral load (median, IQR)	4.35 × 10^5^ [5.85 × 10^4^ - 1.25 × 10^7^]	2.37 × 10^5^ [0 - 1.05 × 10^7^]	2.80 × 10^5^ [3.58 × 10^4^–1.20 × 10^7^]
DENV IgG	1/17 (5.9%)	24/62 (39%)	25/79 (32%)
ZIKV IgG	0/6 (0%)	7/60 (12%)	7/66 (11%)
DENV&ZIKV IgG	0/6 (0%)	5/59 (8.5%)	5/65 (7.7%)
*Analysis 1: Whole-genome sequencing*			
*n*	3	21	24
Days since symptom onset at inclusion (median, IQR)	1 [1; 1] (1 N/A)	2 [1; 4] (2 N/A)	2 [1; 4] (3 N/A)
WHO 2009 classification [2]			
NS	3/3 (100%)	15/21 (71%)	18/24 (75%)
S	0	5/21 (24%)	5/24 (21%)
N/A	0	1/21 (5%)	1/24 (4%)
Infecting serotype			
DENV-1	3/3 (100%)	21/21 (100%)	24/24 (100%)
DENV-2	0	0	0
DENV-3	0	0	0
DENV-4	0	0	0
Viral load (median, IQR)	1.32 × 10^7^ [1.28 × 10^7^ - 2.44 × 10^7^]	2.95 × 10^7^ [9.12 × 10^5^–1.40 × 10^8^]	2.95 × 10^7^ [1.06 × 10^6^–1.29 × 10^8^]
DENV IgG	0/3 (0%)	3/19 (16%)	3/22 (14%)
ZIKV IgG	0/3 (0%)	2/19 (11%)	2/22 (9.1%)
DENV&ZIKV IgG	0/3 (0%)	1/18 (5.6%)	1/21 (4.8%)
*Analysis 2: Kinetics of replication in human hepatic cells*			
*n*	6	7	13
Days since symptom onset at inclusion (median, IQR)	2 [0; 2.5] (2 N/A)	2 [1.5; 2]	2 [1; 2] (2 N/A)
WHO 2009 classification [2]			
NS	2	6	8/13 (62%)
S	0	0	0/13
N/A	4	1	5/13 (38%)
Infecting serotype			
DENV-1	6	7	13/13 (100%)
DENV-2	0	0	0
DENV-3	0	0	0
DENV-4	0	0	0
Viral load (median, IQR)	2.44 × 10^7^ [7.78 × 10^6^–1.08 × 10^8^]	6.89 × 10^7^ [2.95 × 10^7^–1.39 × 10^8^]	3.56 × 10^7^ [2.33 × 10^7^–1.32 × 10^8^]
DENV IgG	0/6 (0%)	1/7 (14%)	1/13 (7.7%)
ZIKV IgG	0/1	0/7	0/8
DENV&ZIKV IgG	0/1	0/7	0/8

Dengue patients are characterized according to the WHO 2009 criteria [2, 40]. DENV serotype and viral load were determined by qRT-PCR. NS, non-severe; S, severe; N/A, not available.

Due to limitations in the amount of biological material available and medical records documentation, biological and clinical characteristics were available for only part of the cohort. Severity criteria were documented in 15/17 (88.2%) severe patients in 2016-2017: the most frequent were thrombocytopenia/bleeding (9/15, 60% severe forms); AST/ALT > 1,000 UI/L (7/15, 47% severe forms); And acute hepatitis (7/15, 47% severe forms) (Supplementary figure 1). Among the latter, three fulminant hepatitis were reported, two of which were fatal. Hepatic dysfunction (ALT/AST > 10 N) was documented in 21/54 (39%) AST/ALT-tested patients in 2016-2017.

DENV-1 was the major infecting serotype, causing 14/17 (82%) and 53/69 (77%) of investigated cases in 2013–2014 and 2016-2017, respectively. Viral loads in 2013–2014 and 2016–2017 samples were comparable, with a median of 4.35 × 10^5^ RNA copies/mL (IQR [5.85 × 10^4^–1.25 × 10^7^]) in 2013–2014 and 2.37 × 10^5^ RNA copies/mL (IQR [0–1.05 × 10^7^]) in 2016–2017 (*p* = 0.23).

Anteriority of dengue or Zika infection was determined by the detection of anti-DENV or ZIKV IgG by ELISA. Secondary infections (presence of anti-DENV IgG) were detected in 1/17 (5.9%) and 24/62 (39%) patients in 2013–2014 and 2016-2017, respectively. Anti-ZIKV IgG were found in 0/17 (0%) and 7/60 (12%) patients from the successive epidemics. Among those anti-ZIKV IgG positive, 5/6 (83.3%) had both anti-ZIKV and DENV IgG.

**Figure 1.**
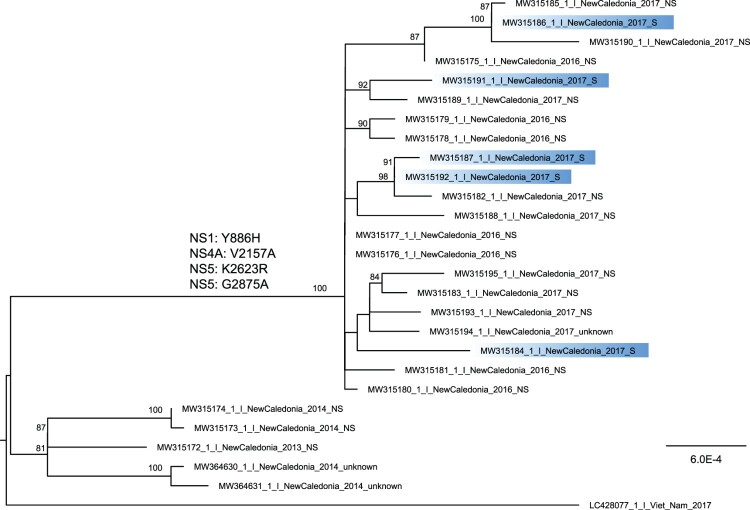
**Phylogenetic tree of DENV-1 whole-genome sequences retrieved from dengue patients sera from 2013**–**2014 and 2016**–**2017.** Whole-genome sequences retrieved from severe forms are highlighted in blue. The tree is midpoint rooted and bootstrap values above 80% are indicated. Two DENV-1 genomes from strains retrieved in NC in 2014 are displayed on the tree. The four amino acid substitutions detected in strains from 2016–2017 in comparison to strains from 2013–2014 are indicated.

### Emergence of a new cluster of viruses in 2016–2017

Among the 86 samples, DENV-1 whole-genome could be sequenced for 3/17 (17.6%) samples from 2013 to 2014 and 21/69 (30.4%) from 2016 to 2017. Whole genomes were successfully sequenced for samples with the highest viremia (Table 1). Sequences obtained for 2013–2014 cases were retrieved from non severe cases. In 2016–2017 among the 21 whole-genomes, 15 (71%) cases were non severe, 5 (24%) were severe and one was not documented clinically. To place these new viral sequences in context, we inferred a phylogeny using all near complete genomes retrieved from the ViPR database (Supplementary figure 2). The sequences reported here formed a monophyletic clade when including a sequence from French Polynesia (bootstrap score 100%) and two sequences from strains retrieved in NC in 2014, within genotype I. We built another phylogeny using only the sequences reported here, with the two sequences from strains retrieved in NC in 2014 and the closest outgroup (Figure 1).

**Figure 2.**
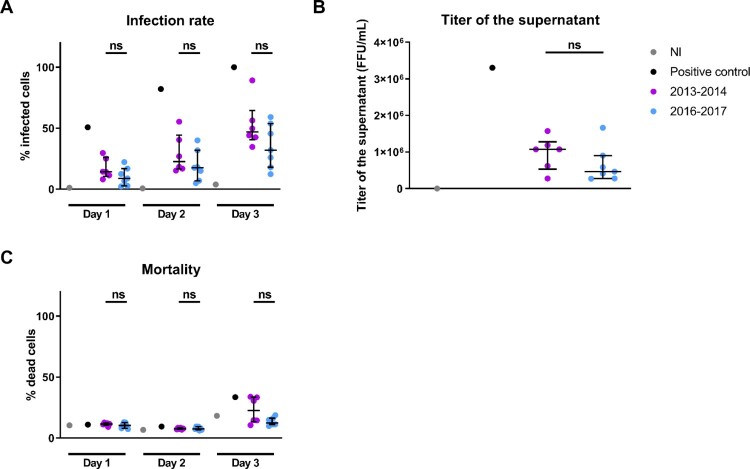
**Replication kinetics of DENV-1 viral isolates from 2013**–**2014 and 2016–2017 in the human hepatic cell line HuH7.** A DENV strain isolated in NC in 2014 passaged several times in C6/36 cells and yielding a high infection rate at day 3 post-infection was used as positive control. Human hepatic HuH7 cells were infected at a MOI of 1 with DENV-1 from 2013–2014 and 2016–2017. A. Percentages of HuH7 cells staining positive for 2H2 antibody measured by flow cytometry at days 1, 2 and 3 post-infection. Percentages of infected cells are normalized by the percentage of infected cells of the positive control at day 3 post-infection. B. Productivity of the infection measured as the viral titer in the supernatant of HuH7 at day 2 post-infection. C. Percentage of dead HuH7 cells staining positive for Viability dye measured by flow cytometry at days 1, 2 and 3 post-infection. NI: non-infected. Each dot is the mean of three independent replicates. Medians and interquartile ranges are indicated. ns: non significant difference in Wilcoxon-Mann-Whitney ranksum test.

Although DENV-1 from 2016 to 2017 were related to viruses from 2013 to 2014, they formed a distinct cluster compared to 2013–2014 viruses (Figure 1). DENV-1 from 2016 to 2017 all shared 26 synonymous mutations and four mutations resulting in amino acid substitutions in non-structural proteins which were absent from the 2013–2014 viruses (Supplementary table 1). A histidine replaced a tyrosine at position 113 in NS1 (NS1 Y886H). An alanine replaced a valine at position 64 in NS4A (NS4A V2157A). Finally, an arginine replaced a lysine at position 132 (NS5 K2623R) and an alanine replaced a glycine at position 384 (NS5 G2875A) in NS5. Residues 887, 2158 and 2871 are located in T cell epitopes as predicted using NetCTL 1.2 server. Residues 870–894 belong to a B cell epitope as predicted using BepiPred 2.0. Viruses sequenced from severe and non-severe 2016–2017 cases were interspersed in the phylogenetic tree.

### Isolated viruses from 2016 to 2017 tend to be less infectious in human hepatic cells than viruses from 2013 to 2014

We successfully isolated six DENV-1 from 2013 to 2014 and seven DENV-1 from 2016 to 2017 contained in patients’ sera through inoculation of C6/36 cells (Table 1). Viral fitness of these strains from 2013–2014 and 2016–2017 were compared in kinetics of replication in HuH7. Although differences in the percentage of infected cells at days 1, 2 and 3 post-infection yielded by viruses from 2013–2014 and 2016–2017 were not statistically significant, viral isolates from 2016–2017 showed a twice lower median percentage of infected cells at day 3 post-infection as compared to viral isolates from 2013–2014 (Figure 2A). Differences in infection productivity as measured by the titer of infectious viral particles in the supernatant at day 2 post-infection was not statistically significant between viruses from 2013–2014 and 2016–2017 (Figure 2B).

### Isolated viruses from 2016 to 2017 tend to be less cytopathogenic for human hepatic cells than viruses from 2013 to 2014

The rate of cell death increased at day 3 post-infection for all DENV strains. Although differences in the percentage of dead cells at days 1, 2 and 3 post-infection yielded by viruses from 2013–2014 and 2016–2017 were not statistically significant, the median percentage of dead cells at day 3 post-infection yielded by viral isolates from 2016 to 2017 was twice lower than the one yielded by viruses from 2013 to 2014 (Figure 2C).

## Discussion

Dengue 2016–2017 outbreak in New Caledonia was characterized by an increase in the percentage of severe forms reported, associated to an unusually high rate of hepatic dysfunctions.

Our study shows that DENV-1 strains from 2016 to 2017 form a phylogenetic cluster distinct but linked to strains retrieved during the previous outbreak, which occurred in 2013-2014. This suggests continuous DENV-1 circulation associated with a genetic evolution between 2013–2014 and 2016-2017. This DENV-1 lineage was not identified in NC in 2015, suggesting that DENV-1 continuous circulation between 2013–2014 and 2016–2017 may have occurred undetected. Alternatively, DENV-1 circulation in NC in 2016–2017 could result from subsequent introduction from a region where a phylogenetically related DENV-1 strain was circulating. Although recurrent homotypic dengue outbreaks are often associated to genotype/clade/lineage displacements [21–24], persistence of the same DENV lineage over a decade or more has been reported elsewhere [21, 23, 25–27].

In our study, the 5 DENV-1 strains retrieved from severe forms in 2016–2017 did not display characteristic genetic features differentiating them from strains retrieved from 15 non-severe dengue cases. This suggests the absence of genetic specificity for strains causing severe dengue in our sample set. Others have shown a specific genetic signature for DENV strains according to the related clinical presentation. A study conducted on DENV strains isolated in Cambodia in 2007 found that a DENV strain isolated from a patient experiencing Dengue Shock Syndrome (DSS) differed from DENV strains isolated from patients experiencing Dengue Fever (DF) or Dengue Hemorrhagic Fever (DHF) by six amino acids, with mutations located in the pre-membrane gene, the envelope gene, and the non-structural (NS) 1, NS3 and NS5 genes [28].

In our study, however, the 21 DENV-1 strains from 2016 to 2017 shared 26 synonymous mutations and four mutations resulting in amino acid substitutions undetected in the five DENV-1 strains from 2013 to 2014. These mutations may have affected protein functions and interactions. The replacement of the polar residue tyrosine at position 113 in NS1 by a polar and basic histidine (NS1 Y886H) [29] occurred in the disordered loop of NS1 α/β wing domain [30]. This substitution may have affected both NS1 acid dissociation constant (pKa) and its tridimensional structure, therefore possibly affecting its protein–protein interactions as well as its function. Although NS4A V2157A, NS5 K2623R and NS5 G2875A led to the substitution of amino acids displaying the same biochemical properties as the original amino acids, these mutations could alter protein localization and function, ultimately impacting DENV-1 phenotype.

Most importantly, these mutations may have modified the antigenicity of DENV-1 accessory proteins: NS1 Y886H mutation occurred near a T cell epitope as predicted using NetCTL server 1.2 and within a B cell epitope as predicted using BepiPred 2.0, and may have affected NS1 antigenicity. NS1 immunization leads to immune-mediated liver injury in mice and passive immunization with anti-NS1 IgG from DENV patients leads to an increase in ALT/AST levels in mice [31]. The NS1 Y886H mutation may thus have modified NS1 antigenicity and contributed to an enhanced NS1-mediated liver injury. Similarly, NS4A V2157A and NS5 G2875A occurred in the vicinity of T cell epitopes. Amino-acid substitutions in DENV accessory proteins have been reported to sustain the attenuation of DENV in the South Pacific region in the 1970s, suggesting that genetic modifications may condition dengue epidemic severity [32]. Single amino acid changes have been reported to lead to drastic phenotypic changes for other viruses: A single amino acid change E1-A226V affected chikungunya virus vector specificity and is considered to have modified its epidemic potential [33]. Similarly, Ebola virus variant A82V in the glycoprotein (GP) exhibited delays in the replication in insectivorous bat Tb1.Lu cells [34]. The enhanced severity of DENV-1 2016–2017 outbreak may thus have been determined by an altered antigenicity of DENV-1 accessory proteins, affecting the host adaptive immune response to DENV infection.

The observed mutations shared by 2016–2017 strains did not significantly modify their ability to replicate in our cellular model of human hepatic cell line as compared to strains from 2013–2014. Some mutations, especially occurring in the E gene, have been associated to an enhanced ability of DENV strains to replicate either in Monocyte-Derived Macrophages (MDM) [35] or in dendritic cells (DC) [36]. Modifications in the secondary structures of the 5’ and 3’ untranslated region also alter the viral output in MDM and DC [36]. We observed, however, a trend towards a less efficient replication of strains from 2016–2017 as compared to strains from the previous outbreak. Interestingly, DENV-1 became the minority serotype in 2018 in NC, being gradually replaced by DENV-2 [37] and was undetected in 2019-2020. This decrease in viral fitness may have contributed to DENV-1 decline in 2018.

Despite a trend towards reduced cytotoxicity for strains retrieved in 2016-2017, the non-synonymous mutations shared by 2016–2017 strains did not significantly alter their ability to trigger cell death in human hepatic cells HuH7. Other studies have explored the link between the clinical presentation of DENV infection and the cytopathogenicity of the corresponding DENV strain. Tuiskunen et al. [28] found that a DENV strain isolated from a DSS patient induced enhanced apoptosis in an *in vitro* model of mosquito C6/36 cells compared to strains isolated from DF or DHF patients. Similarly, DENV-2 strains isolated from non-severe and severe forms of dengue showed different cytopathic effects in C6/36 cells [38]. Although not observed in our study, DENV cytopathogenicity *in vitro* may therefore contribute to the severity of dengue clinical presentation.

Finally, the occurrence of severe dengue in 2016–2017 may have been conditioned by host-related factors as well. Consistent with the study by Marois et al. [7], secondary dengue infection showed a tendency to be associated to a higher risk of developing severe dengue (OR 2.847, CI95 [0.88; 9.2]).

Our study suffers from biases and limitations.

First, a limitation to our study is the missing clinico-epidemiological data for some patients whose samples were included. Documented data, however, are consistent with the data collected by Marois et al. [7], strengthening their reliability.

Second is the low number of whole-genome sequences and viral isolates retrieved from our initial sample set of 86 serum samples. Such low yield results from the comparatively low viral load in these serum samples, which can be due to viral and RNA degradation occurring between sample collection and biobanking. Such sample degradation unfortunately often occurs in studies dealing with human samples as the workload of diagnosis laboratories inexorably increases during dengue outbreaks. Nonetheless, the number of strains challenged in our *in vitro* replication model is comparable to other studies [39]. Further, the trends we observed may have been confirmed would a higher number of strains have been sequenced and isolated.

Third, although HuH7 cells have been used by others in similar *in vitro* replication studies, this cell line may not represent the best cellular model for our study as it may have affected the measured infectivity and cytopathogenicity of isolated strains. The effects we measured could be challenged in other human hepatic cellular models such as HepG2 cells.

Fourth, although no specific genetic features were characteristic of viruses sequenced from severe forms, we were unable to isolate and test viruses from severe forms in our cellular model. Challenging the replicative fitness and cytopathogenicity of viruses isolated from severe forms would have been informative.

Fifth and finally, we decided to focus our study on DENV-1, which was the major circulating serotype in 2016-2017. Severe forms, hepatic presentations and death occurred in DENV-2 and DENV-3 cases as well although to a lesser extent. Although severity seemed to be independent from the incriminated serotype [7], most severe cases were DENV-1 + . Further, the DENV-2 strain circulating in NC in 2017 as minority serotype pursued its circulation in 2018–2019 [37]. The lethality in 2018 was however lower in 2018 as compared to 2017 (0.1% in 2018 VS 0.3% in 2017) [6] and the rate of hepatic presentations decreased in 2018 according to clinicians.

In conclusion, with a hospital admission rate of 8.2%, an important rate of hepatic presentations and 15 deaths, dengue 2016–2017 outbreak in NC was of enhanced severity as compared to previous years. Although DENV strains involved in severe forms did not display a specific genetic signature, dengue 2016–2017 outbreak in NC was characterized by the evolution of the DENV-1 strain as compared to the strain circulating during the previous 2013–2014 outbreak. This viral evolution was not associated to a statistically significant modification in virus replication or cytopathogenicity in our cellular model of human hepatic cells, perhaps due to low number of strains tested. The increase in the number of severe cases during dengue 2016–2017 outbreak in NC is probably not determined by the virus only but rather by host-related factors, history of previous arboviral infection and virus-host interactions. Finally, the contribution of DENV-1 molecular evolution in 2016–2017 on DENV-1 extinction in 2018 in NC remains to be investigated.

## Supplementary Material

SevereDengueViralEvolution-SupFig2-1_editable.jpg
